# Ozonized Hydrogel and Chlorhexidine Gel for Peri‐Implant Mucositis: A 24‐Month Randomized Controlled Trial

**DOI:** 10.1111/odi.70120

**Published:** 2025-10-13

**Authors:** Andrea Scribante, Maurizio Pascadopoli, Matteo Pellegrini, Marco Saturnino Lupi, Carlos Pérez‐Albacete Martínez, Andrea Butera

**Affiliations:** ^1^ Unit of Orthodontics and Pediatric Dentistry, Section of Dentistry, Department of Clinical, Surgical, Diagnostic and Pediatric Sciences University of Pavia Pavia Italy; ^2^ Section of Dentistry, Department of Clinical, Surgical, Diagnostic and Pediatric Sciences University of Pavia Pavia Italy; ^3^ Department of Biomedical, Surgical and Dental Sciences University of Milan Milan Italy; ^4^ Maxilo‐Facial Surgery and Dental Unit Fondazione IRCCS Cà Granda Ospedale Maggiore Policlinico Milan Italy; ^5^ Tissue Regeneration and Repair Group, Biomaterials and Tissue Engineering, Faculty of Health Sciences UCAM‐Universidad Catòlica San Antonio de Murcia Murcia Spain; ^6^ Unit of Dental Hygiene, Section of Dentistry, Department of Clinical, Surgical, Diagnostic and Pediatric Sciences University of Pavia Pavia Italy

**Keywords:** chlorhexidine, clinical outcomes, non‐surgical therapy, ozonated gel, peri‐implant mucositis, split‐mouth randomized controlled trial

## Abstract

**Objective:**

This randomized controlled trial evaluated the 24‐month effectiveness of a nonsurgical intervention for peri‐implant mucositis, comparing a 15% ozonated sunflower oil hydrogel with a 1% chlorhexidine gel.

**Methods:**

Thirty patients with 360 peri‐implant mucositis sites were treated in a split‐mouth design, receiving both therapies in randomly assigned quadrants. Clinical parameters—probing pocket depth (PPD), plaque index (PI), bleeding on probing (BoP), bleeding score (BS), suppuration index (SI), and marginal mucosal condition (MMC)—were recorded at baseline and nine follow‐ups. Marginal bone level (MBL) was measured radiographically at baseline, 6, 15, and 24 months. Recurrence was defined as the reappearance of BoP, SI, or MMC after resolution. Data were analyzed with the Friedman test and Dunn's post hoc.

**Results:**

Both treatments produced significant improvements. The ozonated hydrogel achieved faster and greater reductions in BoP, BS, and SI, with significant differences at 6 and 24 months. MBL remained stable in both groups, with no progression to peri‐implantitis. No adverse events occurred, and compliance was maintained.

**Conclusions:**

The ozonated sunflower hydrogel provided superior anti‐inflammatory effects over chlorhexidine, particularly in reducing bleeding and suppuration, while preserving bone stability. Its safety, efficacy, and acceptability support its use as an adjunct in peri‐implant mucositis maintenance.

## 1 Introduction

Dental implants are widely recognized as a dependable treatment option for restoring function and esthetics in individuals with partial or complete edentulism, demonstrating consistent long‐term success (Atsuta et al. 2015). However, despite their high rates of survival, biological complications—most notably peri‐implant mucositis and peri‐implantitis—continue to pose significant clinical challenges (Gobbato et al. 2013).

Peri‐implant mucositis is defined as the presence of bleeding on gentle probing, with or without suppuration, in the absence of radiographic bone loss beyond the initial physiological remodeling following implant placement. Probing depths may be increased compared with previous examinations, but without evidence of progressive bone loss (Tonetti et al. 2023). Clinically, peri‐implant mucositis is frequently associated with visible signs of inflammation of the peri‐implant mucosa, including erythema, edema, swelling, and an increase in peri‐implant sulcular or crevicular fluid volume; these manifestations reflect the underlying inflammatory infiltrate and remain fully reversible when identified early and managed with appropriate non‐surgical therapy (Herrera, Beglundh, et al. 2023; Iorio‐Siciliano et al. 2024). If not properly addressed, this condition may evolve into peri‐implantitis, characterized by continuing bone resorption, and reported to affect between 28% and 56% of patients and 12% to 43% of implant sites (Laine et al. 2006).

The pathogenesis of peri‐implant diseases is multifactorial, arising from the interaction between microbial imbalance (dysbiosis) and the host's inflammatory and immune responses. A pathogenic shift in the biofilm composition is often marked by an increase in anaerobic Gram‐negative bacteria, particularly those belonging to the orange and red complexes, including *Fusobacterium nucleatum* , *Porphyromonas gingivalis* , and *Tannerella forsythia* (Herrera, Retamal‐Valdes, et al. 2023). Several systemic and local risk factors contribute to disease onset and progression, such as a history of periodontitis, smoking, metabolic conditions like diabetes, poor oral hygiene, inadequate keratinized mucosa, iatrogenic factors, and excess cement (Korsch et al. 2014; Renvert et al. 2018; Lang et al. 2011; Kozlovsky et al. 2007; Mombelli et al. 2018).

Conventional nonsurgical management of peri‐implant mucositis typically involves mechanical biofilm removal with ultrasonic devices or instruments equipped with PEEK tips, frequently combined with antimicrobial therapies (Ince et al. 2015). Chlorhexidine (CHX), due to its strong substantivity and broad antimicrobial spectrum, is commonly considered the benchmark agent in this context (Lindhe et al. 1975). Nevertheless, prolonged administration of CHX is limited by several adverse effects, such as tooth staining, disturbances in taste perception, and irritation of the oral mucosa (Burns et al. 1997). Additionally, its rapid affinity for salivary proteins and the possible development of microbial resistance further compromise its long‐term applicability (Laugisch et al. 2022). In line with this, the Consensus Report by Amodeo et al. (Amodeo et al. 2023) highlighted that CHX, while useful as a supportive measure, does not provide substantial clinical improvement in outcomes such as BoP or PPD when used as an adjunct alone—consistent with the limited intergroup differences observed in the control group of this study.

Ozone (O_3_) has gained attention as a novel adjunct in periodontal and peri‐implant therapies (Nogales et al. 2008; Gandhi et al. 2019; Yu et al. 2022; Li et al. 2013). This triatomic oxygen molecule exhibits potent antimicrobial, anti‐inflammatory, and immunomodulatory effects through mechanisms involving microbial cell wall disruption, oxidation of nucleic acids and proteins, and viral inactivation (Gandhi et al. 2019; Li et al. 2013). In dentistry, ozone is used in various delivery forms—gaseous, aqueous, and oil‐based—and has been applied in caries prevention, endodontic disinfection, periodontal debridement, and implant surface decontamination (Nogales et al. 2008; Yu et al. 2022). In particular, ozonated sunflower oil‐based hydrogel provides a biocompatible and stable platform for the sustained release of active oxygen species.

Despite its promising biological properties, clinical data supporting the long‐term efficacy of ozone‐based formulations in peri‐implant mucositis are still limited. For instance, a randomized controlled trial by Choudhary and Rajasekar (2024) evaluated ozonated olive oil gel over a four‐week period, reporting improvements in plaque and gingival indices compared to CHX. However, the short duration, limited outcome parameters, and absence of recurrence assessment limit the broader applicability of these findings. Such gaps underscore the need for well‐designed trials with longer follow‐up periods and more comprehensive clinical endpoints.

Current research is committed to the evaluation of the best adjunctive treatment for peri‐implant mucositis, suggesting that patient self‐administered antiseptics and probiotics as an adjunct to peri‐implant mucositis treatment may be beneficial (Herrera, Berglundh, et al. 2023). In this context, the present randomized controlled clinical trial was conducted to compare the long‐term clinical outcomes of a 15% ozonated sunflower oil‐based hydrogel versus a 1% CHX gel in the non‐surgical treatment of peri‐implant mucositis. The analysis focused on key clinical variables including Probing Pocket Depth (PPD), Plaque Index (PI), Bleeding on Probing (BoP), Bleeding Score (BS), Suppuration Index (SI), and Marginal Mucosal Condition (MMC). A split‐mouth design and a 24‐month follow‐up period were employed to evaluate the potential benefits of the adjunctive use of ozonated gel, both professionally and at home, in reducing clinical signs of inflammation and minimizing recurrence risk.

Two null hypotheses were established for this study. The first posited that there would be no significant differences between the 15% ozonated sunflower oil‐based hydrogel and the 1% CHX gel in improving peri‐implant clinical parameters—including Probing Pocket Depth (PPD), Plaque Index (PI), Bleeding on Probing (BoP), Bleeding Score (BS), Suppuration Index (SI), and Marginal Mucosal Condition (MMC)—over a 24‐month period. The second hypothesis assumed that the use of ozonated sunflower hydrogel would not lead to a lower recurrence rate of peri‐implant mucositis when compared to CHX gel during the same timeframe.

## 2 Materials and Methods

### 2.1 Study Design

A split‐mouth randomized controlled design was adopted for this clinical trial. The research protocol received approval from both the Unit Internal Review Board (approval ID: 2021‐1201) and the Ethics Committee of Universidad Católica San Antonio (approval ID: CE032212). The trial was prospectively registered on ClinicalTrials.gov under the identifier NCT05254275.

The research adhered to the ethical standards outlined in the Declaration of Helsinki for studies involving human participants and complied with the CONSORT 2025 checklist criteria (Hopewell et al. 2025) (supporting information Table S1). All individuals provided written informed consent prior to their inclusion in the study.

### 2.2 Participants

Participant recruitment, clinical evaluations, and data collection were all conducted at the Dental Hygiene Unit, Section of Dentistry, Department of Clinical, Surgical, Diagnostic, and Pediatric Sciences, University of Pavia, 27100 Pavia, Italy.

#### 2.2.1 Eligibility Criteria for Participants

To obtain a homogeneous study sample, well‐defined inclusion and exclusion criteria were implemented. Participants had to be adults (≥ 18 years) in overall good health, presenting with a minimum of two dental implants diagnosed with peri‐implant mucositis. The diagnosis was based on the presence of bleeding on gentle probing and/or suppuration, accompanied by visible clinical signs of inflammation—such as erythema and edema—without radiographic evidence of bone loss beyond the physiological post‐placement remodeling phase (Herrera, Begrlundh, et al. 2023).

All implants were evaluated clinically and radiographically at baseline to confirm the absence of peri‐implantitis. Periapical radiographs were taken using a standardized long‐cone paralleling technique to verify that no marginal bone loss beyond the initial physiological remodeling had occurred. Only implants meeting this criterion were included in the study.

Only patients with implants located in both the upper and lower arches were considered to allow for the application of a split‐mouth study design. Additional inclusion criteria included the absence of peri‐implant treatment in the previous 6 months and good compliance with oral hygiene instructions, as well as the willingness to attend scheduled follow‐up visits.

Exclusion criteria comprised current smoking or the use of tobacco products, pregnancy or breastfeeding, and the presence of systemic conditions that could affect periodontal or peri‐implant health (such as uncontrolled diabetes or immunodeficiencies). Patients undergoing or having recently completed antibiotic, anti‐inflammatory, or corticosteroid therapy were excluded, as were individuals with a history of peri‐implantitis or known allergies to any components of the tested gels. Poor compliance with oral hygiene or study instructions also constituted a criterion for exclusion.

### 2.3 Interventions

#### 2.3.1 Clinical Procedure and Outcomes

All clinical procedures adhered to the previously defined inclusion and exclusion criteria. At baseline (T0), a calibrated examiner performed clinical assessments using a UNC‐15 periodontal probe (Hu‐Friedy, Chicago, IL, USA). Evaluations were conducted at both the patient and implant levels, recording measurements at six sites per implant (mesiobuccal, buccal, distobuccal, mesiolingual, lingual, and distolingual) affected by peri‐implant mucositis. The following clinical parameters were collected: Probing Pocket Depth (PPD) (AbdulAzeez and Alkinani 2021), Plaque Index (PI) (Silness and Löe 1964), Bleeding on Probing (BoP) (Doornewaard et al. 2018), Bleeding Score (BS) (Mombelli et al. 1987), Suppuration Index (SI) (Butera et al. 2023), and Marginal Mucosal Condition (MMC) (Apse et al. 1991), defined as a clinical index scored on a 0–3 scale to qualitatively assess peri‐implant marginal mucosa. A score of 0 indicates healthy mucosa with normal color and contour; 1 corresponds to mild erythema and/or slight swelling; 2 reflects moderate inflammation with pronounced erythema, swelling, and changes in surface texture; and 3 denotes severe inflammation with marked redness, edema, ulceration, and/or spontaneous bleeding.

Periapical radiographs were obtained at baseline (T0) using a standardized long‐cone paralleling technique to confirm the absence of marginal bone loss beyond the initial physiological remodeling, thereby excluding peri‐implantitis. The same radiographic protocol was repeated only at the scheduled follow‐up time points (T3, T6, and T9) to assess longitudinal changes and calculate variations in marginal bone level (MBL) relative to baseline.

The primary outcome was the change in BoP from baseline (T0) to each follow‐up time point, assessed both at implant level and patient level. Secondary outcomes included changes in PPD, PI, BS, SI, and MMC across the same time points.

Recurrence was operationally defined as the reappearance of at least one clinical sign of mucositis (BoP > 0, SI > 0, or MMC ≥ 1) at a previously resolved site. A site was considered resolved if it met the most recent EFP S3‐Level Clinical Practice Guidelines criteria (Herrera, Begrlundh, et al. 2023), namely BoP ≤ 1, absence of suppuration (SI = 0), and absence of other clinical signs of inflammation (MMC = 0) at the previous time point. Recurrence monitoring was carried out longitudinally from T3 to T9 to assess the long‐term stability of clinical resolution.

For the analysis at the patient level, mean values for probing depth (PD), bleeding score (BS), suppuration index (SI), and marginal mucosal condition (MMC) were obtained by averaging all measurements recorded across peri‐implant sites within each participant. In contrast, plaque index (PI) and bleeding on probing (BoP) were expressed as percentages, indicating the proportion of implant sites exhibiting detectable plaque or bleeding relative to the total number of sites assessed per patient (Bagnasco et al. 2024).

Clinical parameters at the implant level were assessed at six circumferential sites around each implant (mesiobuccal, buccal, distobuccal, mesiolingual, lingual, and distolingual). The Plaque Index (PI) was recorded by noting the presence or absence of plaque at each site, while Bleeding on Probing (BoP) was scored as positive when bleeding occurred within 30 s after gentle probing with a calibrated periodontal instrument (Apaza‐Bedoya et al. 2024).

Prior to commencing the clinical phase, examiner calibration was conducted using a sample of 10 patients, each exhibiting at least one implant affected by peri‐implant mucositis. Duplicate measurements were taken 48 h apart to assess intra‐examiner reliability. Calibration was considered satisfactory when over 90% of repeated measurements differed by no more than ±1 mm, in accordance with established methodological standards (Mariani et al. 2020).

Nonsurgical mechanical debridement of all mucositis‐affected implants within each patient was then carried out. This included the use of a piezoelectric device (Multipiezo, Mectron S.p.A., Carasco, Italy) and Gracey curettes (Hu‐Friedy, Chicago, IL, USA) for initial scaling. Subgingival debridement was subsequently performed using ultrasonic instruments equipped with titanium and PEEK tips (Implant Cleaning Set S, Mectron S.p.A., Carasco, Italy), along with manual titanium curettes (Implant Curette TIS2CN, Deppeler SA, Rolle, Switzerland). To complete the procedure, biofilm removal was enhanced through air polishing with glycine powder (Mectron S.p.A., Carasco, Italy). The use of glycine powder air polishing was limited to both groups and applied equally according to the supportive care protocol, thereby minimizing any potential bias in the estimation of treatment effects. Furthermore, glycine powder has been shown to exert only a transient effect on peri‐implant inflammation without altering long‐term clinical outcomes, making it unlikely to have masked or amplified the observed differences between interventions.

In line with the split‐mouth design, quadrants Q1 and Q4 were treated with in‐office application of a 1% CHX digluconate gel (Curasept Periodontal Gel, Curaden Healthcare S.p.A., Saronno, Italy), whereas quadrants Q2 and Q3 received a 15% ozonated sunflower oil‐based hydrogel (Ozoral Pro, Innovares S.r.l., Sant'Ilario d'Enza, Italy). The application was carried out in‐office using a syringe with a blunt plastic needle, with the gel left in place for at least 2 min.

For home treatment, patients applied the same gels to the corresponding quadrants once daily for 14 days following each in‐office appointment. Ozoral Gel (Innovares S.r.l., Sant'Ilario d'Enza, Italy) was used at home in the test quadrants, while Curasept Gel was applied in the control quadrants. Patients received verbal and written instructions on how to correctly apply the products, and the appearance and packaging of the gels were differentiated to reduce the risk of misuse.

Follow‐up examinations were conducted at nine timepoints: T1 (1 month), T2 (3 months), T3 (6 months), T4 (9 months), T5 (12 months), T6 (15 months), T7 (18 months), T8 (21 months), and T9 (24 months), during which clinical parameters were re‐evaluated. Standardized periapical radiographs were obtained only at T0, T3, T6, and T9 to monitor MBL. Baseline images (T0) confirmed the absence of bone loss beyond initial remodeling, while the 6‐month evaluation (T3) was chosen to detect possible early pathological changes following treatment. The 15‐month follow‐up (T6) allowed the assessment of medium‐term stability of the peri‐implant bone, bridging the interval between the early phase and the final 24‐month evaluation (T9). This schedule provided a comprehensive overview of bone dynamics over time, ensuring clinically relevant monitoring while limiting unnecessary radiation exposure. The chosen follow‐up schedule differed from the 2–3 month re‐evaluation interval recommended by the EFP S3‐Level Clinical Practice Guidelines for peri‐implant mucositis (Herrera, Begrlundh, et al. 2023). In this study, the T1 visit at 1 month was intentionally included to capture early clinical changes, whereas subsequent long‐term assessments (T3–T9) were designed to monitor the maintenance or recurrence of clinical resolution under supportive peri‐implant care.

At T1 and T2, peri‐implant sites underwent professional decontamination using air‐polishing with glycine powder. Starting from T3 and continuing through T9, each visit included a comprehensive supragingival and subgingival instrumentation session employing sonic or ultrasonic scalers with PEEK tips, along with manual debridement using titanium or PEEK curettes. Decontamination with glycine‐based powder was then repeated. All procedures were performed within a structured supportive periodontal and peri‐implant care protocol.

The composition of the two gels used in the study is detailed in Table 1.

**TABLE 1:** Formulation of the evaluated gels.

Product	Manufacturer	Composition
Ozoral Pro/Ozoral Gel	Innovares S.r.l., Sant'Ilario d'Enza, Italy	Aqua, 15% ozonated sunflower seed oil, aroma, glycerin, carbomer, polycarbophil, sodium hydroxide, sodium saccharin, anise ( *Illicium verum* ) fruit/seed oil, glyceryl caprylate, tocopherol, ascorbyl palmitate, disodium EDTA, limonene, linalool
Curasept Periodontal Gel (with 1% chlorhexidine)	Curaden Healthcare S.p.A., Saronno, Italy	Aqua, Propylene glycol, hydroxyethylcellulose, PVP/VA copolymer, PEG‐40 hydrogenated castor oil, 1% chlorhexidine digluconate, sodium acetate, flavoring, acetic acid, sodium metabisulfite, ascorbic acid

### 2.4 Sample Size Calculation

The sample size calculation was based on a two‐group comparison with a continuous primary outcome (BoP%), using a significance level (*α*) of 0.05 and a statistical power of 95%.

The calculation was performed using the standard formula for comparing means between two groups:

$$\text{Sample size}=4\times \frac{{\left(Z_{\left(1-\frac{\alpha }{2}\right)}+Z_{\left(1-\beta \right)}\right)}^{2}\sigma^{2}}{{∆}^{2}}$$

In the sample size estimation, $Z_{\left(1-\frac{\alpha }{2}\right)}$ corresponds to the critical value from the standard normal distribution for a 5% significance level (1.96), and $Z_{\left(1-\beta \right)}$ corresponds to the value for 95% power (1.645). The parameter σ represents the common standard deviation of BoP% across groups, while ∆ indicates the expected between‐group mean difference.

BoP was designated as the primary outcome measure. Drawing on 1‐month data from a published RCT, the common standard deviation was set to *σ* = 29.83 percentage points and the anticipated difference to *Δ* = 11.29 percentage points (Butera et al. 2023). Given these assumptions, the power analysis determined that a total sample size of 360 peri‐implant mucositis sites would be necessary to achieve adequate statistical power (i.e., 180 sites per arm under 1:1 allocation).

### 2.5 Randomization

A randomization sequence was generated by a biostatistician using permuted block randomization, targeting a total of 360 peri‐implant mucositis sites (180 sites per treatment group). Allocation to treatment was performed in a 1:1 split‐mouth design using a computer‐generated randomization sequence created in R (version 4.4.1; R Foundation for Statistical Computing, Vienna, Austria), ensuring proper allocation concealment. For half of the participants, quadrants Q1 and Q4 were designated as control sites (treated with 1% chlorhexidine gel), while Q2 and Q3 were assigned to the test condition (15% ozonated sunflower oil–based hydrogel); the opposite allocation was applied to the remaining participants. All subjects received standardized instructions for at‐home use of the assigned gels. The randomization sequence was concealed from the clinical investigator and recruitment personnel. The care provider was blinded to the allocation until the moment of treatment administration, thereby ensuring proper allocation concealment.

### 2.6 Blinding and Calibration

For home use, the gel formulations were provided in coded packaging with distinct visual features to support patient compliance while maintaining partial concealment of treatment allocation. Given the characteristics of the intervention, blinding of both the care provider and participants was not feasible. However, the clinical examiner responsible for data collection and outcome evaluation remained blinded to treatment assignments. Additionally, the statistician conducting data analysis was unaware of both group allocation and clinical outcomes throughout the analytical process.

Before initiating the clinical phase, the examiner underwent a calibration process using a cohort of 10 patients, each presenting with at least one implant affected by peri‐implant mucositis. Clinical indices were assessed on two separate occasions, 48 h apart. Calibration was considered acceptable when more than 90% of repeated measurements showed a discrepancy of less than 1 mm, ensuring high intra‐examiner agreement and measurement reliability (Mariani et al. 2020).

### 2.7 Statistical Analysis

Statistical analysis was performed using R software (version 4.4.1; R Foundation for Statistical Computing, Vienna, Austria). Data were analyzed at both the patient and implant levels. The primary inferential analysis was performed at the implant level using paired statistical tests, consistent with the split‐mouth design of the study. Patient‐level analyses were conducted as complementary evaluations to support the clinical interpretation of findings and assess potential trends at the subject level. For each study group, descriptive statistics—means and standard deviations—were calculated for all clinical variables. The Kolmogorov–Smirnov test was used to evaluate the normality of data distributions. Since the data did not follow a normal distribution, non‐parametric methods were applied. Comparisons between groups were conducted using the Kruskal–Wallis test, with Dunn's post hoc test employed for pairwise comparisons. Intra‐group changes across timepoints were assessed with the Friedman test for repeated measures, followed by Dunn's post hoc test where appropriate. A letter‐based grouping method was used to identify statistically significant intergroup and intragroup differences (Piepho 2004). A significance threshold of *p* < 0.05 was established a priori for all statistical analyses.

## 3 Results

### 3.1 Participants and Recruitment

**FIGURE 1:**
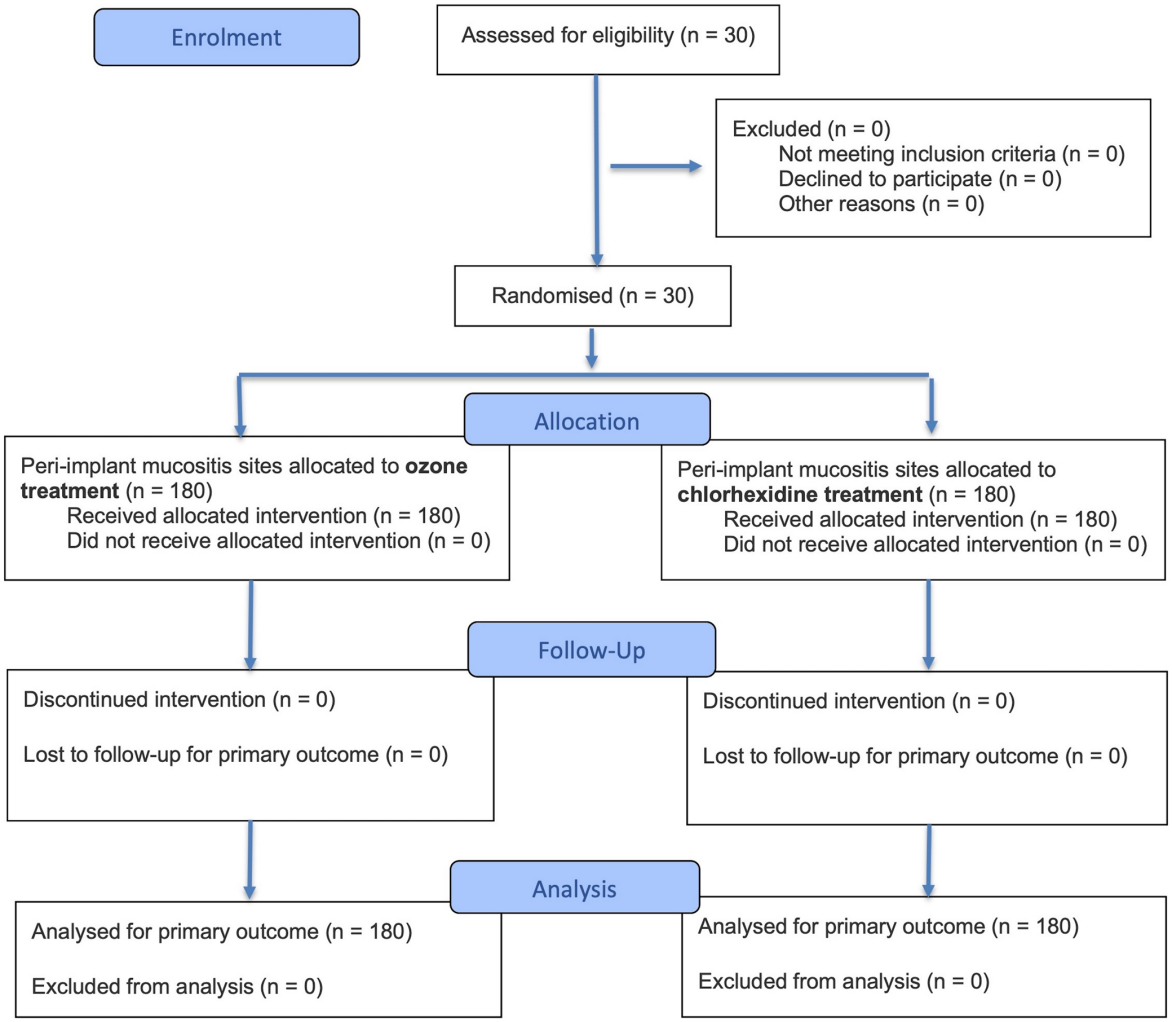
CONSORT 2025 flow diagram.

Figure 1 illustrates the CONSORT 2025 flowchart of the study (Hopewell et al. 2025). Following the screening process, 30 patients who met the eligibility criteria were enrolled at the Dental Hygiene Unit, Section of Dentistry, Department of Clinical, Surgical, Diagnostic and Pediatric Sciences, University of Pavia, Italy. Recruitment was carried out between January 2022 and February 2024. The number of participants was established based on prior sample size estimation, which indicated the necessity of including 180 peri‐implant mucositis sites per group—totaling 360 sites to achieve sufficient statistical power within the split‐mouth framework. No participants discontinued the study, and no adverse events associated with either treatment protocol were observed. In total, 360 peri‐implant mucositis sites were managed: 180 sites received treatment with 1% CHX gel, while the remaining 180 were treated using the ozonated sunflower hydrogel.

### 3.2 Demographic Characteristics

Table 2 summarizes the demographic profile of the study population. A total of 30 individuals were enrolled, comprising 16 males (53.3%) and 14 females (46.7%). The overall mean age was 51.17 ± 10.03 years, with a range of 35 to 77 years. Male participants had a mean age of 53.08 ± 9.99 years (range: 35–68), while the mean age among females was 49.89 ± 10.15 years (range: 38–77). None of the enrolled subjects reported tobacco use, thereby excluding smoking as a confounding variable in the analysis.

**TABLE 2:** Demographic characteristics and implant distribution of sample population.

Variable	Value
Age (years)	
Overall (mean ± SD, range)	51.17 ± 10.03 (35–77)
Male (mean ± SD, range)	53.08 ± 9.99 (35–68)
Female (mean ± SD, range)	49.89 ± 10.15 (38–77)
Gender distribution (*n* [%])	
Male	16 (53.3)
Female	14 (46.7)
Implant position (*n* [%])	
Maxillary	35 (58.33)
Mandibular	25 (41.67)

Central incisor	Lateral incisor	Canine	First premolar	Second premolar	First molar	Second molar
Quadrant 1 (Control group): 16 (26.67)						
1 (1.67)	1 (1.67)	4 (6.67)	4 (6.67)	1 (1.67)	4 (6.67)	1 (1.67)
Quadrant 2 (Test group): 19 (31.67)						
1 (1.67)	1 (1.67)	3 (5.00)	3 (5.00)	4 (6.67)	4 (6.67)	3 (4.76)
Quadrant 3 (Test group): 11 (18.33)						
0 (0)	1 (1.67)	1 (1.67)	2 (3.33)	2 (3.33)	4 (6.67)	1 (1.67)
Quadrant 4 (Control group): 14 (23.33)						
0 (0)	1 (1.67)	1 (1.67)	3 (5.00)	1 (1.67)	4 (6.67)	4 (6.67)

Abbreviation: SD, standard deviation.

### 3.3 Outcomes

In accordance with the split‐mouth design, the primary inferential analyses were conducted at the implant level using paired comparisons between test and control sites within the same patient. Patient‐level results were analyzed as secondary, complementary data to support the clinical interpretation of findings and to provide an overall view of treatment effects within individuals.

Both patient‐level and implant‐level data were analyzed to provide a comprehensive evaluation of peri‐implant tissue conditions. Implant‐level analysis enabled detailed assessment of changes at specific sites, while patient‐level analysis offered a general overview of therapeutic outcomes within individuals.

Significant reductions in PPD (Table 3) over time were observed within both treatment groups (*p* < 0.05). At the implant level, the Test group showed a decrease from 6.82 ± 1.24 mm at baseline (T0) to 2.95 ± 0.94 mm at 24 months (T9), whereas the Control group reduced from 6.55 ± 1.18 mm to 3.72 ± 0.86 mm. Although no statistically significant differences between the groups were found at most time points (*p* > 0.05), the Test group consistently demonstrated lower mean values from T3 onward, reaching statistical significance at T7, T8, and T9. At the patient level, the Test group decreased from 6.71 ± 1.03 mm to 3.01 ± 0.64 mm, while the Control group declined from 6.49 ± 0.91 mm to 3.77 ± 0.73 mm. Again, intergroup comparisons did not yield significant differences (*p* > 0.05).

**TABLE 3:** Descriptive statistics and intragroup/intergroup comparisons of PPD implant‐level and patient‐level values across all time points (T0–T9) in the Test and Control groups.

Group	Time	Mean	SD	Min	Median	Max	Intragroup (T0–T9)*	Intergroup (Control–Test)*
Implant‐level								
Control (*n* = 30)	T0	6.55	1.18	5.00	6.00	10.00	a	A
	T1	5.22	1.17	3.00	5.00	8.00	b	A
	T2	4.49	1.16	2.00	4.00	7.00	bc	A
	T3	4.23	1.13	2.00	4.00	7.00	c	A
	T4	3.98	0.94	2.00	4.00	6.00	cd	A
	T5	3.95	0.92	2.00	4.00	6.00	cd	A
	T6	3.89	0.92	2.00	4.00	6.00	cd	A
	T7	3.81	0.89	2.00	4.00	6.00	cd	A
	T8	3.75	0.87	2.00	4.00	6.00	d	A
	T9	3.72	0.86	2.00	4.00	6.00	d	A
Test (*n* = 30)	T0	6.82	1.24	5.00	7.00	9.00	a	A
	T1	5.42	1.37	3.00	5.00	9.00	b	A
	T2	4.40	1.43	2.00	4.00	9.00	c	A
	T3	3.90	1.46	2.00	4.00	8.00	cd	A
	T4	3.42	1.08	2.00	3.00	7.00	de	A
	T5	3.35	1.05	2.00	3.00	7.00	de	A
	T6	3.28	1.06	2.00	3.00	7.00	de	A
	T7	3.14	1.02	2.00	3.00	6.00	e	B
	T8	3.01	0.94	2.00	3.00	6.00	e	B
	T9	2.95	0.94	1.00	3.00	6.00	e	B
Patient‐level								
Control (*n* = 30)	T0	6.49	0.91	5.00	6.00	9.00	a	A
	T1	5.15	0.89	4.00	5.00	8.00	ab	A
	T2	4.57	1.01	2.00	4.00	6.00	abc	A
	T3	4.30	0.94	2.00	4.00	6.00	abc	A
	T4	4.03	0.75	2.00	4.00	6.00	abc	A
	T5	4.00	0.71	2.00	4.00	6.00	abc	A
	T6	3.92	0.73	2.00	4.00	5.00	c	A
	T7	3.85	0.72	2.00	4.00	5.00	c	A
	T8	3.79	0.73	2.00	4.00	5.00	c	A
	T9	3.77	0.73	2.00	4.00	5.00	c	A
Test (*n* = 30)	T0	6.71	1.03	5.00	7.00	9.00	a	A
	T1	5.23	1.05	4.00	5.00	8.00	b	A
	T2	4.33	1.13	2.00	4.00	8.00	bc	A
	T3	3.89	1.17	2.00	4.00	7.00	cd	A
	T4	3.40	0.80	2.00	3.00	6.00	cd	A
	T5	3.34	0.77	2.00	3.00	6.00	cd	A
	T6	3.29	0.78	2.00	3.00	6.00	cd	A
	T7	3.15	0.75	2.00	3.00	5.00	d	A
	T8	3.05	0.67	2.00	3.00	5.00	d	A
	T9	3.01	0.64	2.00	3.00	5.00	d	A

*Note:* Intragroup analysis considered all the time frames of the study for the comparisons (lowercase letters), while intergroup analysis considered only Control versus Trial comparison at each time frame (uppercase letters).

Abbreviations: Max, maximum; Min, minimum; SD, standard deviation; T0, baseline; T1, 1 month; T2, 3 months; T3, 6 months; T4, 9 months; T5, 12 months; T6, 15 months; T7, 18 months; T8, 21 months; T9, 24 months.

* Means presenting at least one identical letter are not significantly different (*p* > 0.05).

Both groups experienced significant intragroup reductions in PI over time (*p* < 0.05; Table 4). At the implant level, the Test group dropped from 1.00 ± 0.00 at T0 to 0.04 ± 0.20 at T9, while the Control group decreased from 1.00 ± 0.00 to 0.30 ± 0.46. Although the Test group exhibited greater numerical improvement, no statistically significant intergroup differences were found up to T6 (*p* > 0.05). From T7 onward, however, the Test group achieved significantly lower values compared to the Control group (*p* < 0.05). At the patient level, the Test group declined from 84.27% ± 16.24% to 21.20% ± 14.02%, while the Control group reduced from 84.90% ± 16.70% to 37.30% ± 14.40%. Intergroup comparisons confirmed the superior performance of the Test group at T7, T8, and T9 (*p* < 0.05).

**TABLE 4:** Descriptive statistics and intragroup/intergroup comparisons of PI implant‐level and patient‐level values across all time points (T0–T9) in the Test and Control groups.

Group	Time	Mean	SD	Min	Median	Max	Intragroup (T0–T9)*	Intergroup (Control–Test)*
Implant‐level								
Control (*n* = 30)	T0	1.00	0.00	1.00	1.00	1.00	a	A
	T1	0.95	0.22	0.00	1.00	1.00	ab	A
	T2	0.97	0.17	0.00	1.00	1.00	a	A
	T3	0.96	0.20	0.00	1.00	1.00	ab	A
	T4	0.97	0.17	0.00	1.00	1.00	a	A
	T5	0.86	0.35	0.00	1.00	1.00	ab	A
	T6	0.63	0.49	0.00	1.00	1.00	bc	A
	T7	0.40	0.49	0.00	0.00	1.00	cd	A
	T8	0.26	0.44	0.00	0.00	1.00	d	A
	T9	0.30	0.46	0.00	0.00	1.00	cd	A
Test (*n* = 30)	T0	1.00	0.00	1.00	1.00	1.00	a	A
	T1	0.96	0.20	0.00	1.00	1.00	abc	A
	T2	0.97	0.17	0.00	1.00	1.00	a	A
	T3	0.97	0.17	0.00	1.00	1.00	abc	A
	T4	0.97	0.17	0.00	1.00	1.00	ab	A
	T5	0.88	0.34	0.00	1.00	1.00	ab	A
	T6	0.65	0.48	0.00	1.00	1.00	bc	A
	T7	0.25	0.43	0.00	0.00	1.00	d	A
	T8	0.08	0.28	0.00	0.00	1.00	d	A
	T9	0.04	0.20	0.00	0.00	1.00	d	A
Patient‐level								
Control (*n* = 30)	T0	84.90	16.70	45.00	89.50	100.00	a	A
	T1	49.90	1.80	20.00	46.50	100.00	bcd	A
	T2	50.10	16.50	15.00	46.50	88.00	acd	A
	T3	47.20	19.40	15.00	42.00	81.00	acd	A
	T4	42.90	18.30	10.00	36.50	77.00	de	A
	T5	41.40	17.40	10.00	38.00	75.00	de	A
	T6	40.10	16.60	10.00	37.50	70.00	de	A
	T7	40.30	15.30	10.00	40.00	67.00	de	A
	T8	38.40	13.90	10.00	38.50	60.00	e	A
	T9	37.30	14.40	10.00	33.50	62.00	e	A
Test (*n* = 30)	T0	84.27	16.24	45.00	87.50	100.00	a	A
	T1	45.63	17.05	10.00	43.00	88.00	b	A
	T2	45.10	18.23	10.00	43.00	79.00	b	A
	T3	42.03	21.13	0.00	41.00	83.00	b	A
	T4	36.07	19.41	5.00	36.00	76.00	bc	A
	T5	32.70	16.86	5.00	33.50	70.00	cd	A
	T6	29.50	16.21	5.00	29.50	73.00	cd	A
	T7	25.93	15.35	1.00	28.00	72.00	cd	B
	T8	23.73	14.86	0.00	23.50	68.00	d	B
	T9	21.20	14.02	0.00	20.00	65.00	d	B

*Note:* Intragroup analysis considered all the time frames of the study for the comparisons (lowercase letters), while intergroup analysis considered only Control versus Trial comparison at each time frame (uppercase letters).

Abbreviations: Max, maximum; Min, minimum; SD, standard deviation; T0, baseline; T1, 1 month; T2, 3 months; T3, 6 months; T4, 9 months; T5, 12 months; T6, 15 months; T7, 18 months; T8, 21 months; T9, 24 months.

* Means presenting at least one identical letter are not significantly different (*p* > 0.05).

Significant reductions in BoP (Table 5) were observed over time in both groups (*p* < 0.05). At the implant level, the Test group decreased from 1.00 ± 0.00 at T0 to 0.00 ± 0.00 at T9, while the Control group declined from 1.00 ± 0.00 to 0.11 ± 0.32. Although no significant intergroup differences were found up to T8 (*p* > 0.05), a statistically significant advantage for the Test group emerged at T9 (*p* < 0.05). At the patient level, BoP decreased from 46.14% ± 27.09% to 3.33% ± 4.01% in the Test group and from 44.60% ± 26.90% to 13.30% ± 8.46% in the Control group. From T5 onward, intergroup comparisons consistently favored the Test group, reaching statistical significance (*p* < 0.05).

**TABLE 5:** Descriptive statistics and intragroup/intergroup comparisons of BoP implant‐level values across all time points (T0–T9) in the Test and Control groups.

Group	Time	Mean	SD	Min	Median	Max	Intragroup (T0–T9)*	Intergroup (Control–Test)*
Implant‐level								
Control (*n* = 30)	T0	1.00	0.00	1.00	1.00	1.00	a	A
	T1	0.98	0.14	0.00	1.00	1.00	ab	A
	T2	0.97	0.17	0.00	1.00	1.00	a	A
	T3	0.97	0.17	0.00	1.00	1.00	ab	A
	T4	0.96	0.20	0.00	1.00	1.00	a	A
	T5	0.88	0.33	0.00	1.00	1.00	ab	A
	T6	0.72	0.45	0.00	1.00	1.00	bc	A
	T7	0.48	0.50	0.00	0.00	1.00	cd	A
	T8	0.29	0.46	0.00	0.00	1.00	d	A
	T9	0.11	0.32	0.00	0.00	1.00	cd	A
Test (*n* = 30)	T0	1.00	0.00	1.00	1.00	1.00	a	A
	T1	0.96	0.20	0.00	1.00	1.00	ab	A
	T2	0.97	0.17	0.00	1.00	1.00	ab	A
	T3	0.97	0.17	0.00	1.00	1.00	ab	A
	T4	0.96	0.20	0.00	1.00	1.00	ab	A
	T5	0.85	0.37	0.00	1.00	1.00	ab	A
	T6	0.47	0.50	0.00	0.00	1.00	bc	A
	T7	0.19	0.39	0.00	0.00	1.00	d	A
	T8	0.01	0.10	0.00	0.00	1.00	d	A
	T9	0.00	0.00	0.00	0.00	0.00	d	A
Patient‐level								
Control (*n* = 30)	T0	44.60	26.90	8.33	44.00	100.00	a	A
	T1	27.10	17.20	0.00	23.70	55.00	ab	A
	T2	18.40	12.30	1.19	19.30	40.30	abc	A
	T3	16.10	10.20	1.10	16.50	36.00	bc	A
	T4	14.10	9.00	1.00	15.00	33.00	bc	A
	T5	13.90	8.73	1.00	15.00	32.00	bc	A
	T6	13.80	9.05	1.00	11.50	35.00	bc	A
	T7	13.40	8.76	0.00	11.00	35.00	bc	A
	T8	13.20	8.38	0.00	12.50	30.00	c	A
	T9	13.30	8.46	0.00	12.50	31.00	bc	A
Test (*n* = 30)	T0	46.14	27.09	5.95	42.50	100.00	a	A
	T1	21.73	13.66	0.00	21.00	48.00	ab	A
	T2	14.08	9.15	0.00	13.50	37.10	bc	A
	T3	10.78	7.72	0.00	10.10	29.40	bcd	A
	T4	7.60	6.61	0.00	5.00	25.00	cde	A
	T5	5.80	5.49	0.00	5.00	20.00	de	B
	T6	4.95	5.49	0.00	3.50	22.50	de	B
	T7	4.40	4.87	0.00	3.50	20.00	e	B
	T8	3.57	4.25	0.00	2.50	18.00	e	B
	T9	3.33	4.01	0.00	2.00	15.00	e	B

*Note:* Intragroup analysis considered all the time frames of the study for the comparisons (lowercase letters), while intergroup analysis considered only Control versus Trial comparison at each time frame (uppercase letters).

Abbreviations: Max, maximum; Min, minimum; SD, standard deviation; T0, baseline; T1, 1 month; T2, 3 months; T3, 6 months; T4, 9 months; T5, 12 months; T6, 15 months; T7, 18 months; T8, 21 months; T9, 24 months.

* Means presenting at least one identical letter are not significantly different (*p* > 0.05).

Statistically significant reductions in BS were observed in both groups over time (*p* < 0.05; Table 6). At the implant level, the Test group decreased from 2.07 ± 0.70 at baseline (T0) to 0.07 ± 0.25 at 24 months (T9), while the Control group declined from 2.13 ± 0.66 to 0.53 ± 0.50. Intergroup comparisons revealed significant differences at T3 and from T6 through T9, consistently favoring the Test group (*p* < 0.05). At the patient level, BS values fell from 2.14 ± 0.61 to 0.06 ± 0.16 in the Test group, and from 2.21 ± 0.58 to 0.61 ± 0.40 in the Control group. Statistically significant intergroup differences emerged from T6 onward, again favoring the Test group (*p* < 0.05).

**TABLE 6:** Descriptive statistics and intragroup/intergroup comparisons of BS implant‐level and patient‐level values across all time points (T0–T9) in the Test and Control groups.

Group	Time	Mean	SD	Min	Median	Max	Intragroup (T0–T9)*	Intergroup (Control–Test)*
Implant‐level								
Control (*n* = 30)	T0	2.13	0.66	1.00	2.00	3.00	a	A
	T1	1.81	0.75	0.00	2.00	3.00	ab	A
	T2	1.50	0.76	0.00	2.00	3.00	ab	A
	T3	1.31	0.68	0.00	1.00	2.00	bc	A
	T4	0.95	0.46	0.00	1.00	2.00	cd	A
	T5	0.91	0.47	0.00	1.00	2.00	cd	A
	T6	0.80	0.49	0.00	1.00	2.00	d	A
	T7	0.68	0.52	0.00	1.00	2.00	d	A
	T8	0.59	0.55	0.00	1.00	2.00	d	A
	T9	0.53	0.50	0.00	1.00	1.00	d	A
Test (*n* = 30)	T0	2.07	0.70	1.00	2.00	3.00	a	A
	T1	1.56	0.74	0.00	2.00	3.00	ab	A
	T2	1.19	0.71	0.00	1.00	3.00	bc	A
	T3	0.87	0.68	0.00	1.00	2.00	cd	B
	T4	0.63	0.49	0.00	1.00	1.00	de	A
	T5	0.53	0.50	0.00	1.00	1.00	def	A
	T6	0.37	0.49	0.00	0.00	1.00	ef	B
	T7	0.25	0.44	0.00	0.00	1.00	fg	B
	T8	0.13	0.34	0.00	0.00	1.00	fg	B
	T9	0.07	0.25	0.00	0.00	1.00	g	B
Patient‐level								
Control (*n* = 30)	T0	2.21	0.58	1.00	2.00	3.00	a	A
	T1	1.70	0.82	0.00	2.00	3.00	ab	A
	T2	1.44	0.65	0.00	1.50	2.33	abc	A
	T3	1.19	0.55	0.00	1.25	2.00	abcd	A
	T4	0.92	0.42	0.00	1.00	2.00	bcd	A
	T5	0.89	0.41	0.00	1.00	2.00	bcd	A
	T6	0.83	0.44	0.00	1.00	2.00	cd	A
	T7	0.74	0.45	0.00	0.88	2.00	d	A
	T8	0.68	0.48	0.00	0.75	2.00	d	A
	T9	0.61	0.40	0.00	0.75	1.00	d	A
Test (*n* = 30)	T0	2.14	0.61	1.00	2.00	3.00	a	A
	T1	1.49	0.73	0.00	1.50	3.00	ab	A
	T2	1.15	0.60	0.00	1.00	3.00	abc	A
	T3	0.82	0.53	0.00	1.00	2.00	bcd	A
	T4	0.59	0.37	0.00	0.71	1.00	cde	A
	T5	0.50	0.37	0.00	0.58	1.00	de	A
	T6	0.36	0.37	0.00	0.25	1.00	de	A
	T7	0.26	0.35	0.00	0.00	1.00	de	A
	T8	0.11	0.22	0.00	0.00	1.00	e	A
	T9	0.06	0.16	0.00	0.00	1.00	e	A

*Note:* Intragroup analysis considered all the time frames of the study for the comparisons (lowercase letters), while intergroup analysis considered only Control versus Trial comparison at each time frame (uppercase letters).

Abbreviations: Max, maximum; Min, minimum; SD, standard deviation; T0, baseline; T1, 1 month; T2, 3 months; T3, 6 months; T4, 9 months; T5, 12 months; T6, 15 months; T7, 18 months; T8, 21 months; T9, 24 months.

* Means presenting at least one identical letter are not significantly different (*p* > 0.05).

Regarding SI, significant reductions were likewise recorded over time (*p* < 0.05; Table 7). At the implant level, the Test group decreased from 0.17 ± 0.38 at baseline (T0) to 0.00 ± 0.00 at 24 months (T9), while the Control group declined from 0.16 ± 0.37 to 0.00 ± 0.00. Although intragroup changes were statistically significant (*p* < 0.05), no intergroup differences emerged at any time point (*p* > 0.05). At the patient level, SI values dropped from 0.19 ± 0.36 to 0.00 ± 0.00 in the Test group, and from 0.20 ± 0.36 to 0.00 ± 0.00 in the Control group. Complete elimination of suppuration was achieved by T5 in the Test group and by T4 in the Control group, with results maintained through T9. Intergroup comparisons did not reveal significant differences (*p* > 0.05).

**TABLE 7:** Descriptive statistics and intragroup/intergroup comparisons of SI implant‐level and patient‐level values across all time points (T0–T9) in the Test and Control groups.

Group	Time	Mean	SD	Min	Median	Max	Intragroup (T0–T9)*	Intergroup (Control–Test)*
Implant‐level								
Control (*n* = 30)	T0	0.16	0.37	0.00	0.00	1.00	a	A
	T1	0.13	0.34	0.00	0.00	1.00	ab	A
	T2	0.05	0.23	0.00	0.00	1.00	ab	A
	T3	0.04	0.20	0.00	0.00	1.00	bc	A
	T4	0.00	0.00	0.00	0.00	0.00	c	A
	T5	0.00	0.00	0.00	0.00	0.00	c	A
	T6	0.00	0.00	0.00	0.00	0.00	c	A
	T7	0.00	0.00	0.00	0.00	0.00	c	A
	T8	0.00	0.00	0.00	0.00	0.00	c	A
	T9	0.00	0.00	0.00	0.00	0.00	c	A
Test (*n* = 30)	T0	0.17	0.38	0.00	0.00	1.00	a	A
	T1	0.08	0.27	0.00	0.00	1.00	ab	A
	T2	0.05	0.22	0.00	0.00	1.00	b	A
	T3	0.03	0.16	0.00	0.00	1.00	b	A
	T4	0.01	0.11	0.00	0.00	1.00	b	A
	T5	0.00	0.00	0.00	0.00	0.00	b	A
	T6	0.00	0.00	0.00	0.00	0.00	b	A
	T7	0.00	0.00	0.00	0.00	0.00	b	A
	T8	0.00	0.00	0.00	0.00	0.00	b	A
	T9	0.00	0.00	0.00	0.00	0.00	b	A
Patient‐level								
Control (*n* = 30)	T0	0.20	0.36	0.00	0.00	1.00	a	A
	T1	0.16	0.33	0.00	0.00	1.00	a	A
	T2	0.08	0.25	0.00	0.00	1.00	a	A
	T3	0.07	0.24	0.00	0.00	1.00	a	A
	T4	0.00	0.00	0.00	0.00	0.00	b	A
	T5	0.00	0.00	0.00	0.00	0.00	b	A
	T6	0.00	0.00	0.00	0.00	0.00	b	A
	T7	0.00	0.00	0.00	0.00	0.00	b	A
	T8	0.00	0.00	0.00	0.00	0.00	b	A
	T9	0.00	0.00	0.00	0.00	0.00	b	A
Test (*n* = 30)	T0	0.19	0.36	0.00	0.00	1.00	a	A
	T1	0.09	0.22	0.00	0.00	1.00	ab	A
	T2	0.06	0.20	0.00	0.00	1.00	ab	A
	T3	0.05	0.19	0.00	0.00	1.00	b	A
	T4	0.03	0.17	0.00	0.00	1.00	b	A
	T5	0.00	0.00	0.00	0.00	0.00	b	A
	T6	0.00	0.00	0.00	0.00	0.00	b	A
	T7	0.00	0.00	0.00	0.00	0.00	b	A
	T8	0.00	0.00	0.00	0.00	0.00	b	A
	T9	0.00	0.00	0.00	0.00	0.00	b	A

*Note:* Intragroup analysis considered all the time frames of the study for the comparisons (lowercase letters), while intergroup analysis considered only Control versus Trial comparison at each time frame (uppercase letters).

Abbreviations: Max, maximum; Min, minimum; SD, standard deviation; T0, baseline; T1, 1 month; T2, 3 months; T3, 6 months; T4, 9 months; T5, 12 months; T6, 15 months; T7, 18 months; T8, 21 months; T9, 24 months.

* Means presenting at least one identical letter are not significantly different (*p* > 0.05).

Improvements in MMC also followed a significant trajectory in both groups (*p* < 0.05; Table 8). At the implant level, the Test group decreased from 2.29 ± 0.48 at baseline (T0) to 0.03 ± 0.16 at 24 months (T9), while the Control group reduced from 2.29 ± 0.49 to 0.43 ± 0.50. From T7 onward, the Test group consistently showed more favorable mean values, with statistically significant intergroup differences detected at T7 and T8 (*p* < 0.05). At the patient level, the Test group improved from 2.27 ± 0.42 to 0.05 ± 0.20, while the Control group declined from 2.26 ± 0.43 to 0.57 ± 0.43. Intergroup comparisons reached significance from T4 through T9 (*p* < 0.05), consistently favoring the Test group.

**TABLE 8:** Descriptive statistics and intragroup/intergroup comparisons of MMC implant‐level and patient‐level values across all time points (T0–T9) in the Test and Control groups.

Group	Time	Mean	SD	Min	Median	Max	Intragroup (T0–T9)*	Intergroup (Control–Test)*
Implant‐level								
Control (*n* = 30)	T0	2.29	0.49	1.00	2.00	3.00	a	A
	T1	1.63	0.75	0.00	2.00	3.00	b	A
	T2	1.32	0.60	0.00	1.00	2.00	bc	A
	T3	1.09	0.68	0.00	1.00	2.00	bcd	A
	T4	0.88	0.59	0.00	1.00	2.00	cde	A
	T5	0.81	0.56	0.00	1.00	2.00	def	A
	T6	0.72	0.48	0.00	1.00	2.00	def	A
	T7	0.59	0.50	0.00	1.00	1.00	ef	A
	T8	0.49	0.50	0.00	0.00	1.00	ef	A
	T9	0.43	0.50	0.00	0.00	1.00	f	A
Test (*n* = 30)	T0	2.29	0.48	1.00	2.00	3.00	a	A
	T1	1.62	0.73	1.00	1.00	3.00	b	A
	T2	1.23	0.65	0.00	1.00	2.00	bc	A
	T3	1.00	0.73	0.00	1.00	2.00	c	A
	T4	0.53	0.53	0.00	1.00	2.00	de	A
	T5	0.43	0.50	0.00	0.00	1.00	def	A
	T6	0.36	0.48	0.00	0.00	1.00	def	A
	T7	0.14	0.35	0.00	0.00	1.00	ef	B
	T8	0.06	0.25	0.00	0.00	1.00	f	B
	T9	0.03	0.16	0.00	0.00	1.00	f	A
Patient‐level								
Control (*n* = 30)	T0	2.26	0.43	1.00	2.25	3.00	a	A
	T1	1.59	0.73	0.00	1.75	3.00	ab	A
	T2	1.33	0.47	0.00	1.25	2.00	abc	A
	T3	1.11	0.52	0.00	1.00	2.00	b	A
	T4	0.96	0.50	0.00	1.00	2.00	bcd	A
	T5	0.89	0.45	0.00	1.00	2.00	bcd	A
	T6	0.77	0.31	0.00	1.00	1.00	cd	A
	T7	0.68	0.37	0.00	0.75	1.00	d	A
	T8	0.62	0.39	0.00	0.71	1.00	d	A
	T9	0.57	0.43	0.00	0.67	1.00	d	A
Test (*n* = 30)	T0	2.27	0.42	1.00	2.25	3.00	a	A
	T1	1.60	0.54	1.00	1.50	3.00	ab	A
	T2	1.21	0.49	0.00	1.25	2.00	ab	A
	T3	0.99	0.56	0.00	1.00	2.00	bc	A
	T4	0.52	0.43	0.00	0.63	1.00	cd	A
	T5	0.42	0.42	0.00	0.38	1.00	cd	A
	T6	0.34	0.37	0.00	0.25	1.00	d	A
	T7	0.14	0.27	0.00	0.00	1.00	d	A
	T8	0.08	0.21	0.00	0.00	1.00	d	A
	T9	0.05	0.20	0.00	0.00	1.00	d	A

*Note:* Intragroup analysis considered all the time frames of the study for the comparisons (lowercase letters), while intergroup analysis considered only Control versus Trial comparison at each time frame (uppercase letters).

Abbreviations: Max, maximum; Min, minimum; SD, standard deviation; T0, baseline; T1, 1 month; T2, 3 months; T3, 6 months; T4, 9 months; T5, 12 months; T6, 15 months; T7, 18 months; T8, 21 months; T9, 24 months.

* Means presenting at least one identical letter are not significantly different (*p* > 0.05).

At baseline (T0), the mean MBL (Table 9) was 1.30 ± 0.92 mm in the Control group and 1.21 ± 0.69 mm in the Test group. Over the 24‐month follow‐up, both groups maintained stable values without clinically relevant bone loss beyond initial remodeling. Specifically, in the Control group, MBL remained unchanged, measuring 1.30 ± 0.91 mm at T3, 1.29 ± 0.92 mm at T6, and 1.29 ± 0.91 mm at T9. In the Test group, a slight numerical reduction was observed, from 1.19 ± 0.69 mm at T3 to 1.14 ± 0.66 mm at T6, reaching 1.12 ± 0.64 mm at T9. The inferential analysis with the Kruskal–Wallis test confirmed the absence of statistically significant differences both between groups and across time points (*H* = 1.094; *p* = 0.9932).

**TABLE 9:** Marginal bone level (mm) at baseline and follow‐up.

Group	Time	Mean	SD	Min	Median	Max
Control (*n* = 30)	T0	1.30	0.92	0.00	1.00	4.50
	T3	1.30	0.91	0.00	1.00	4.50
	T6	1.29	0.92	0.00	1.00	4.50
	T9	1.29	0.91	0.00	1.00	4.50
Test (*n* = 30)	T0	1.21	0.69	0.00	1.00	3.50
	T3	1.19	0.69	0.00	1.00	3.50
	T6	1.14	0.66	0.00	1.00	3.50
	T9	1.12	0.64	0.00	1.00	3.50

According to the stringent EFP S3‐level (Herrera, Begrlundh, et al. 2023) endpoint definition (absence of suppuration and BoP ≤ 1 point), only 1 implant per group (1.67%) achieved clinical resolution at 3 months (T2), whereas all other implants failed to reach this therapeutic target and required further intervention.

## 4 Discussion

This randomized, split‐mouth controlled trial assessed the long‐term clinical efficacy of a 15% ozonated sunflower oil‐based hydrogel compared to a 1% CHX gel in the nonsurgical treatment of peri‐implant mucositis. Over the 24‐month follow‐up, both interventions yielded notable clinical improvements; however, the ozonated sunflower oil hydrogel demonstrated superior performance in reducing specific inflammatory markers and suppuration. These results support the rejection of both null hypotheses, confirming the presence of statistically significant differences between groups and suggesting that the adjunctive use of the ozonated oil‐based gel contributes to enhanced clinical resolution of mucositis. Although recurrence was not assessed as a formal outcome, clinical monitoring throughout the study indicated sustained suppression of BoP, SI, and MMC in the Test group from T5 to T9, pointing to a reduced risk of relapse and improved long‐term stability. Notably, when evaluated against the stringent EFP S3‐level (Herrera, Begrlundh, et al. 2023) endpoint definition (absence of suppuration and BoP ≤ 1 point), only 1 implant per group achieved clinical resolution at 3 months (T2). This underscores the difficulty of attaining complete remission in the short term and justifies the extended follow‐up adopted in our protocol, which allowed for the capture of progressive improvements and long‐term stability beyond the early healing phase.

Both treatment groups showed significant reductions in PPD over time, reinforcing the effectiveness of nonsurgical debridement paired with antiseptic adjuncts. The reductions were consistent at both patient and implant levels, highlighting a comprehensive therapeutic response. While intergroup differences did not reach statistical significance, the Test group demonstrated consistently lower mean PPD values starting from T3, indicating a possible supplementary advantage associated with the use of the ozonated sunflower oil hydrogel. These findings are consistent with previous studies in which ozonated oil‐based interventions produced deeper and more sustained probing depth reductions (Isler et al. 2018; Scribante et al. 2022; Hayakumo et al. 2013). The oxidative and immunomodulatory properties of ozonated oil may enhance soft tissue healing within the peri‐implant sulcus, even in the absence of statistically significant group separation. In vitro studies have shown that ozone can effectively reduce peri‐implant pathogens such as *P. gingivalis* adhered to titanium and zirconia surfaces without altering surface morphology or impairing osteoblast‐like cell adhesion and proliferation (Hauser‐Gerspach et al. 2012). Moreover, the observation that adjunctive ozone therapy after mechanical debridement yields additional PPD reduction and CAL gain in periodontitis patients supports its applicability in peri‐implant disease management (Isler et al. 2021).

PI improved significantly in both groups across all time points. At the implant level, the Test group approached complete plaque elimination by T9, while at the patient level, it demonstrated a more pronounced reduction, although without reaching statistical significance compared to the Control. These observations underscore the role of regular mechanical maintenance and effective oral hygiene in plaque control. Ozonated sunflower oil gels may represent a valid alternative to CHX, offering comparable or potentially superior plaque reduction over the long term, without the limitations associated with CHX, such as protein binding and reduced efficacy over time (Lindhe et al. 1975; Laugisch et al. 2022). The reduction in plaque accumulation observed in our ozonated sunflower oil group is consistent with in vitro findings showing lower bacterial adhesion to zirconia and polished titanium surfaces, particularly when combined with ozonated oil treatment (Hauser‐Gerspach et al. 2012). Furthermore, clinical evidence indicates that the use of ozonated formulations can significantly improve plaque control and gingival health when incorporated into supportive therapy protocols (Swarna Meenakshi and Rajasekar 2022).

BoP emerged as a particularly sensitive indicator of intergroup differences. Although both groups showed significant improvements, only the Test group achieved complete elimination of bleeding at the implant level by T9. At the patient level, statistically significant differences in favor of the Test group were evident from T5 onward. Since BoP is a key diagnostic marker for mucositis (Doornewaard et al. 2018), its sustained suppression suggests a stronger anti‐inflammatory effect associated with the ozonated sunflower oil hydrogel. Previous studies have reported similar benefits when ozonated formulations were incorporated into subgingival protocols (Ince et al. 2015; Hayakumo et al. 2013; Scribante et al. 2024; Kshitish and Laxman 2010), and the present findings expand on this evidence by demonstrating long‐term maintenance of inflammation control. The in vitro observation that ozonated oil can eliminate *P. gingivalis* to below detection limits within 24 s on both titanium and zirconia surfaces (Hauser‐Gerspach et al. 2012) may help explain its pronounced anti‐inflammatory effects in vivo. Additionally, adjunctive ozonated oil therapy has been shown to significantly reduce gingival inflammation in supportive periodontal care, even in sites with persistent BoP (Pereira et al. 2025).

BS, reflecting the severity of bleeding, significantly declined in both groups, with the Test group showing more favorable results. Statistically significant intergroup differences were noted at T3 and T9, suggesting that the ozonated sunflower oil hydrogel may not only reduce bleeding frequency but also attenuate its intensity, potentially through effects on microvascular permeability and local inflammation. These findings align with previous reports on the vascular‐stabilizing properties of ozonated formulations (Colombo et al. 2021; Vasthavi et al. 2020) and with laboratory evidence showing that ozonated oil treatment does not compromise the biocompatibility of implant surfaces for osteoblast‐like cells (Hauser‐Gerspach et al. 2012). Evidence from clinical trials also suggests that the hemostatic effect of ozonated oils may be mediated by their capacity to enhance local tissue oxygenation and reduce inflammatory mediators (Swarna Meenakshi and Rajasekar 2022).

The rapid and sustained reduction in suppuration observed in the Test group further supports the antimicrobial efficacy of the ozonated sunflower oil hydrogel. SI reached zero earlier in the ozonated sites and remained stable through the study's end. In contrast, the Control group showed delayed and less consistent results, with a significant change occurring only at T3. This is in line with the known ability of ozonated oils to disrupt bacterial membranes and inhibit anaerobic biofilms (Gandhi et al. 2019; Li et al. 2013), as corroborated by other clinical studies (Colombo et al. 2021; Tetè et al. 2023) and consensus reports (Amodeo et al. 2023). Moreover, the ability of ozonated formulations to significantly reduce early colonizers such as *S. sanguinis* , even if not completely eradicated in the presence of organic material, may contribute to limiting the microbial succession that favors pathogenic biofilm development (Hauser‐Gerspach et al. 2012). Notably, adjunctive ozonated oil therapy has demonstrated similar antimicrobial persistence in peri‐implant pockets, maintaining reduced anaerobic counts up to several months post‐treatment (D Ambrosio et al. 2023).

Both groups demonstrated significant improvement in MMC scores over time, with the Test group displaying consistently lower values from T4 onward. Although intergroup differences were not statistically significant, the trend favors greater mucosal stability in the ozonated sunflower oil hydrogel group. These results reflect previous findings that link ozonated oil formulations with improved mucosal tone and resilience (Laugisch et al. 2022; Colombo et al. 2021), supporting their inclusion in maintenance protocols for peri‐implant health (Apse et al. 1991). Laboratory evidence that ozonated treatments do not damage implant surfaces while maintaining osteoblast adhesion further supports their suitability for long‐term peri‐implant maintenance (Hauser‐Gerspach et al. 2012). Furthermore, repeated application of ozonated oils in maintenance therapy has been associated with enhanced soft tissue stability and reduced recurrence of peri‐implant inflammation (Pereira et al. 2025).

No adverse events were reported throughout the study, confirming the excellent safety profile of both treatment modalities. These outcomes are consistent with prior studies demonstrating the biocompatibility and non‐cytotoxicity of ozonated oil‐based formulations (Saini 2011; Sechi et al. 2001). High patient adherence and correct home application further support the clinical usability of both gels, a finding consistent with Scribante et al. (Scribante et al. 2024). The absence of adverse effects also mirrors the safety outcomes reported in long‐term supportive care studies using ozonated products as adjuncts (Swarna Meenakshi and Rajasekar 2022; D Ambrosio et al. 2023).

The sustained clinical benefits observed in the ozonated gel group may be attributed to its controlled‐release formulation, which ensures prolonged antimicrobial and anti‐inflammatory activity without the drawbacks associated with CHX, such as staining or altered taste. In contrast to short‐term trials (e.g., Choudhary et al. (Choudhary and Rajasekar 2024)), the present study offers robust long‐term evidence supporting the use of ozonated sunflower oil hydrogel across multiple clinical domains. The results are reinforced by mechanistic evidence from in vitro research showing the ability of ozonated products to selectively reduce peri‐implant bacterial adhesion while preserving surface integrity and cellular compatibility (Hauser‐Gerspach et al. 2012), and by clinical studies demonstrating their sustained benefits in both periodontal and peri‐implant maintenance (Isler et al. 2021; Pereira et al. 2025).

Finally, both treatment groups maintained stable MBL throughout the 24‐month follow‐up, with no signs of progressive bone loss beyond initial remodeling. These results are consistent with the current definition of peri‐implant mucositis as a reversible condition without radiographic bone changes (Herrera, Begrlundh, et al. 2023) and align with previous reports showing that effective nonsurgical management and supportive care can prevent marginal bone loss irrespective of the adjunctive protocol used (Roccuzzo et al. 2022; Ramanauskaite et al. 2021; Taheri et al. 2020).

Nonetheless, some limitations must be acknowledged. Although the split‐mouth design minimized intersubject variability, the possibility of cross‐contamination cannot be completely excluded. The absence of microbiological or immunological assessments limits the ability to directly correlate clinical outcomes with microbial or inflammatory changes. Moreover, the inclusion of only systemically healthy, non‐smoking, and highly compliant participants may restrict the generalizability of the findings. Another limitation is the lack of patient‐reported outcome measures (PROMs), which precludes evaluation of subjective aspects such as taste, comfort, or perceived efficacy.

A further relevant limitation concerns the at‐home application of the gels in the assigned quadrants. While participants were systemically healthy, non‐smoking, and demonstrated high compliance, the study protocol inherently assumed perfect adherence to the 14‐day daily home treatment regimen following each in‐office session. In real‐world settings, however, full compliance cannot be guaranteed, and even minor deviations in frequency, duration, or technique of application could have influenced the magnitude of the treatment effect. Although adherence was verbally reinforced at each visit and clear written instructions were provided, no objective monitoring (e.g., diaries, return of empty syringes, or electronic compliance tracking) was implemented. This lack of verification introduces a potential confounding factor that may have attenuated or amplified the observed intergroup differences and should be considered when interpreting the results.

Furthermore, the inclusion of repeated professional decontamination and supra−/subgingival instrumentation at each follow‐up was an intentional methodological choice, consistent with the EFP S3 Clinical Practice Guidelines for peri‐implant maintenance (Herrera, Begrlundh, et al. 2023). This ensured patient safety, disease control, and adherence to real‐world supportive care protocols, but may have attenuated the measurable differences between the interventions over time. Since the protocol was applied uniformly to both groups, however, any potential confounding effect was equally distributed and did not compromise the study's internal validity.

The follow‐up schedule also deserves consideration. Our design incorporated an early re‐evaluation at 1 month (T1) and subsequent long‐term assessments up to 24 months, which differs from the 2–3 month re‐evaluation interval recommended by the EFP S3‐Level Clinical Practice Guidelines for peri‐implant mucositis (Herrera, Begrlundh, et al. 2023). While this allowed us to capture both short‐term and extended trends under supportive care, it is possible that the earliest assessment (T1) underestimated the treatment effect, whereas later evaluations (T3–T9) may have been more influenced by recurrence dynamics than by the initial intervention. This temporal structure may therefore limit direct comparability with studies strictly adhering to guideline‐based timelines.

Another potential limitation relates to implant position. Although implants were not a priori matched by anatomical site, post hoc analyses confirmed that their distribution among anterior, premolar, and molar regions did not differ significantly between groups, both when analyzed across three categories (*χ* ^2^ = 1.366, df = 2, *p* = 0.505) and when dichotomized into anterior vs. posterior sites (*χ* ^2^ = 0.000, df = 1, *p* = 1.000; Fisher's exact *p* = 1.000). This minimizes the likelihood of positional bias on plaque control–dependent outcomes.

Future studies should integrate microbiological, biochemical, and PROM‐based endpoints to provide a more comprehensive evaluation of ozone therapy. Including patients with systemic comorbidities such as diabetes, or those at higher risk (e.g., smokers), would also enhance external validity. Moreover, comparative trials testing ozone against newer adjuncts (e.g., probiotics, photodynamic therapy, peptide‐based biomodulators) could better define its relative clinical utility. Finally, the potential prophylactic application of ozonated gels in healthy peri‐implant tissues represents an intriguing direction for future research on mucositis prevention.

## 5 Conclusions

In the context of this 24‐month randomized split‐mouth clinical investigation, enhanced clinical outcomes were observed with the use of 15% ozonated sunflower oil‐based hydrogel, when compared to 1% CHX gel, as an adjunct in the nonsurgical treatment of peri‐implant mucositis. Both treatment modalities achieved significant and durable intragroup improvements across all assessed parameters, yet sites treated with ozonized oil hydrogel demonstrated more pronounced reductions in BS and significantly lower BoP values at the patient level from the 12‐month follow‐up (T5) onward. For SI, progressive improvements were observed in both groups without significant intergroup differences, while PPD, PI, and MMC improved comparably, albeit with consistently more favorable trends in the ozonized oil group from the study midpoint. Importantly, MBL remained stable in both groups throughout the 24‐month follow‐up, with no evidence of progression to peri‐implantitis. No adverse reactions were observed, and adherence to both in‐office and domiciliary protocols was generally high; this outcome was supported by the continuous reinforcement and motivation provided at each follow‐up visit, which played a key role in maintaining patient compliance over the two‐year period. Taken together, these results support the incorporation of ozonated sunflower hydrogel as a safe, well‐tolerated, and clinically effective adjunct in the supportive care of patients with peri‐implant mucositis.

## Author Contributions

**Andrea Scribante:** conceptualization, data curation, formal analysis, methodology, project administration, software, supervision, validation, visualization, writing – review and editing. **Maurizio Pascadopoli:** resources, validation, visualization, writing – original draft. **Matteo Pellegrini:** resources, writing – original draft, validation, visualization. **Marco Saturnino Lupi:** conceptualization, methodology, supervision, validation, visualization, writing – review and editing. **Carlos Pérez‐Albacete Martínez:** conceptualization, data curation, formal analysis, methodology, project administration, software, supervision, validation, visualization, writing – review and editing. **Andrea Butera:** conceptualization, project administration, supervision, validation.

## Ethics Statement

The study was conducted in accordance with the ethical standards outlined in the Declaration of Helsinki. Approval was granted by the Internal Review Board of the institution (protocol number 2021‐1201; date of approval: December 1, 2021), and the trial was prospectively recorded on ClinicalTrials.gov under the identifier NCT05254275.

## Consent

Each participant enrolled in the study provided written informed consent prior to inclusion.

## Conflicts of Interest

The authors declare no conflicts of interest.

## Supporting information


**Table S1:** CONSORT 2025 Checklist.

## Data Availability

Data produced and examined within this investigation can be obtained by contacting the corresponding authors, provided the request is justified and appropriate.
